# Highlighting when animals expend excessive energy for travel using dynamic body acceleration

**DOI:** 10.1016/j.isci.2022.105008

**Published:** 2022-08-24

**Authors:** Rory P. Wilson, Samantha D. Reynolds, Jonathan R. Potts, James Redcliffe, Mark Holton, Abi Buxton, Kayleigh Rose, Bradley M. Norman

**Affiliations:** 1 Biosciences, Swansea University, Singleton Park, Swansea SA2 8PP, Wales, UK; 2 School of Biological Sciences, The University of Queensland, St Lucia, QLD 4065, Australia; 3 ECOCEAN Inc., P.O. Box 42, Serpentine, WA 6125, Australia; 4 University of Sheffield, Hicks Building, Hounsfield Road, Sheffield S3 7RH, England, UK; 5 Harry Butler Institute, Murdoch University, Murdoch, WA 6150, Australia

**Keywords:** Biological sciences, biomechanics, biophysics

## Abstract

Travel represents a major cost for many animals so there should be selection pressure for it to be efficient – at minimum cost. However, animals sometimes exceed minimum travel costs for reasons that must be correspondingly important. We use Dynamic Body Acceleration (DBA), an acceleration-based metric, as a proxy for movement-based power, in tandem with vertical velocity (rate of change in depth) in a shark (*Rhincodon typus*) to derive the minimum estimated power required to swim at defined vertical velocities. We show how subtraction of measured DBA from the estimated minimum power for any given vertical velocity provides a “proxy for power above minimum” metric (PPA_min_), highlighting when these animals travel above minimum power. We suggest that the adoption of this metric across species has value in identifying where and when animals are subject to compelling conditions that lead them to deviate from ostensibly judicious energy expenditure.

## Highlights

• Plots of vertical speed vs DBA in sharks show swimming with minimum power
• DBA values above this minimum indicate higher speeds or increases in drag
• Linked to space use, this can identify regions and times of excess power use

## Graphical abstract

**Figure:**
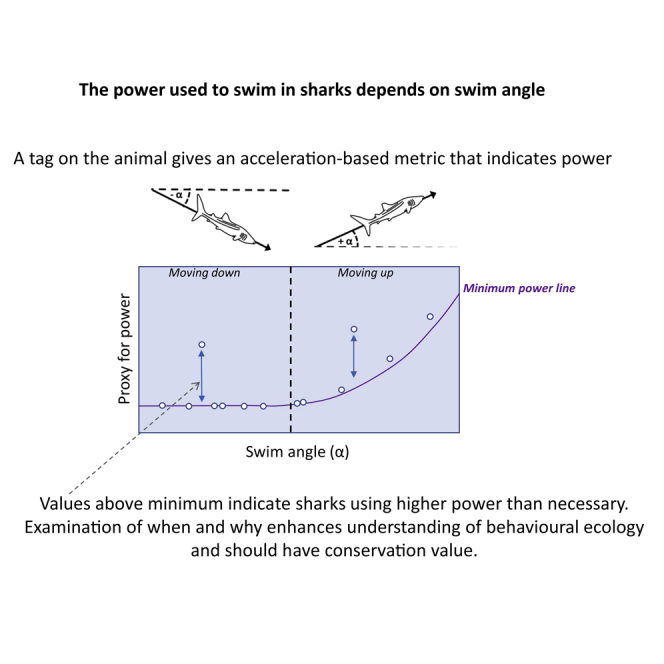

## Introduction

The rate at which animals use energy for activities has received considerable attention because judicious energy expenditure is considered to be a major factor in enhancing lifetime reproductive success (Bright Ross et al., 2020; Pianka, 1976). The cost of travel is a major element in this, with researchers, suggesting that all other things being equal, efficient movement should either be at speeds that result in minimum costs of transport, COT_min_ (Tucker, 1970; Tucker and Catlett, 1973), where the energy cost per unit distance is minimum (Jara Diaz, 1982; Taylor et al., 1982) or, for animals in fluid media, at minimum power, where the energy cost per unit time is minimum (Tucker, 1973). However, maximizing lifetime reproductive success does not always equate to either of these because animals have to increase power for a variety of reasons including the pursuit of prey (Hirt et al., 2020), escape from predators (Bryce et al., 2017), traveling through risky environments (Wheatley et al., 2021) and minimizing time rather than energy e.g., when provisioning young (Shepard et al., 2009). Consequently, identification of times when animals deviate from minimum power costs or COT_min_ has particular value because it should indicate circumstances where power use is higher than expected, presumably because the gains outweigh the energetic inefficiencies. Indeed, being able to identify periods when animals do not adhere to minimum power or COT_min_ during movement, should help clarify specifics in life history strategies and be particularly relevant for species that are considered to be on an energetic knife-edge owing to anthropogenically driven environmental change (Sims, 1999).

The difficulty in determining power-use in animals is that to be conducted properly, it requires the use of gas respirometry on laboratory-confined animals (Kaiyala, 2014; Macfarlane, 2017), significantly limiting the maximum size of animals on which it can be performed (Watanabe and Goldbogen, 2021). But it is particularly onerous where power is to be related to traveling behavior because it is especially difficult to measure oxygen consumption in moving animals (Nelson, 2016). However, dynamic body acceleration (DBA) (Wilson et al., 2020a) has been shown to be a powerful linear predictor of power-use attributed to movement in animals (Gleiss et al., 2010; Halsey et al., 2009; Wilson et al., 2020b; Wright et al., 2014) and it is readily measurable using accelerometers in animal-attached tags (Qasem et al., 2012). DBA is calculated by subtracting the static acceleration component (derived by using a running mean on the raw data (Shepard et al., 2008) of each orthogonal acceleration channel from the raw acceleration to leave the dynamic acceleration components which are then added across channels, either vectorially or simply summed (Wilson et al., 2020a).

We build on the demonstrated value of DBA as a proxy for power (which, however, cannot be equated to the cost of transport unless the speed is known) and propose a method that identifies periods when animals traveling in fluid media (water and air) invest more energy than necessary for minimum power traveling. We use whale sharks *Rhincodon typus* as an example species because sharks are denser than seawater (Craik, 1978; Ste-Marie et al., 2020) and so have an allocation of power used to swim based on their rate of change in depth. Sharks can glide when moving down the water column (e.g., Papastamatiou et al., 2018; Watanabe et al., 2019; cf. Miller et al., 2004) but have to use increasing amounts of power to move with increasingly acute upward angles, which correspond to greater rates of change in depth (Gleiss et al., 2011), even under conditions of minimum power. Our approach thus compares the rate of change in depth against DBA to identify vertical velocity-specific minimum DBA (as a proxy for minimum power) values to derive a functional relationship between them. We note that the minima that make up this functional relationship will also have specific absolute speeds and that where sharks increase this absolute speed but maintain the vertical velocity, their DBA values will leave the vertical velocity-specific minimum DBA line. Thus, our treatment looks for minimum power scenarios rather than, for example, minimum costs of transport, for which measures of speed are needed (Papastamatiou et al., 2018).

We demonstrate how subtracting DBA values from the proxy for minimum power at any given time gives a DBA value at, or above, the minimum (proxy power above minimum - PPA_min_). This metric highlights energetically expensive behavior showing, for example, periods when sharks travel faster than they need for minimum power or when they change their drag coefficient e.g., by opening their mouths to feed.

## Results

Data retrieved from the 12 tag packages deployed on whale sharks covered periods ranging between ∼2.5 h and 21 days.

**Figure 1:**
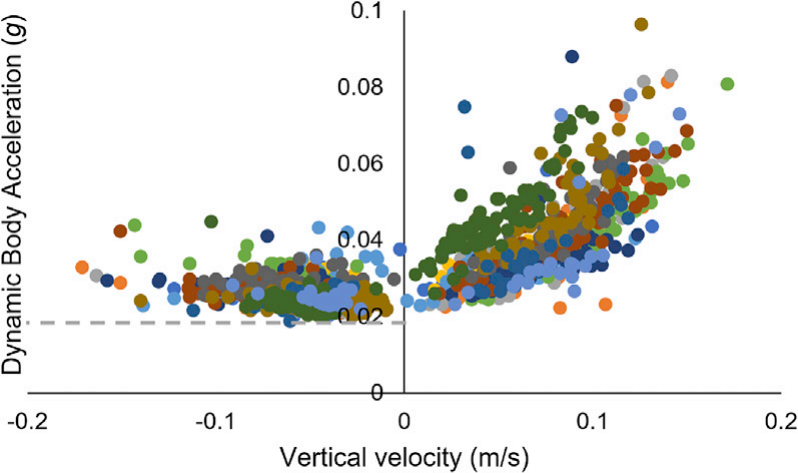
Mean Dynamic Body Acceleration (DBA) Mean Dynamic Body Acceleration (DBA) as a function of mean vertical velocity during steady state movement (each point shows a period of >10 and <50 s, during which the vertical velocity did not vary by more than a maximum of 0.01 m/s) from 12 whale sharks equipped with tags for periods between ∼2.5 h and 21 days. Note, this plot excludes data for depths <3 m and 8 points which either occur at rates of depth change less than −0.2 m/s or which have a DBA >0.1 – which have been omitted here to highlight the detail in the vast majority of the data.

Plots of DBA against vertical velocity typically showed a major distribution of points that increased from negative vertical velocities (sharks descending) to maxima at the highest vertical velocities (sharks ascending) with a lower boundary that was predominantly flat at vertical velocities less than 0 m/s (Figure 1).

**Figure 2:**
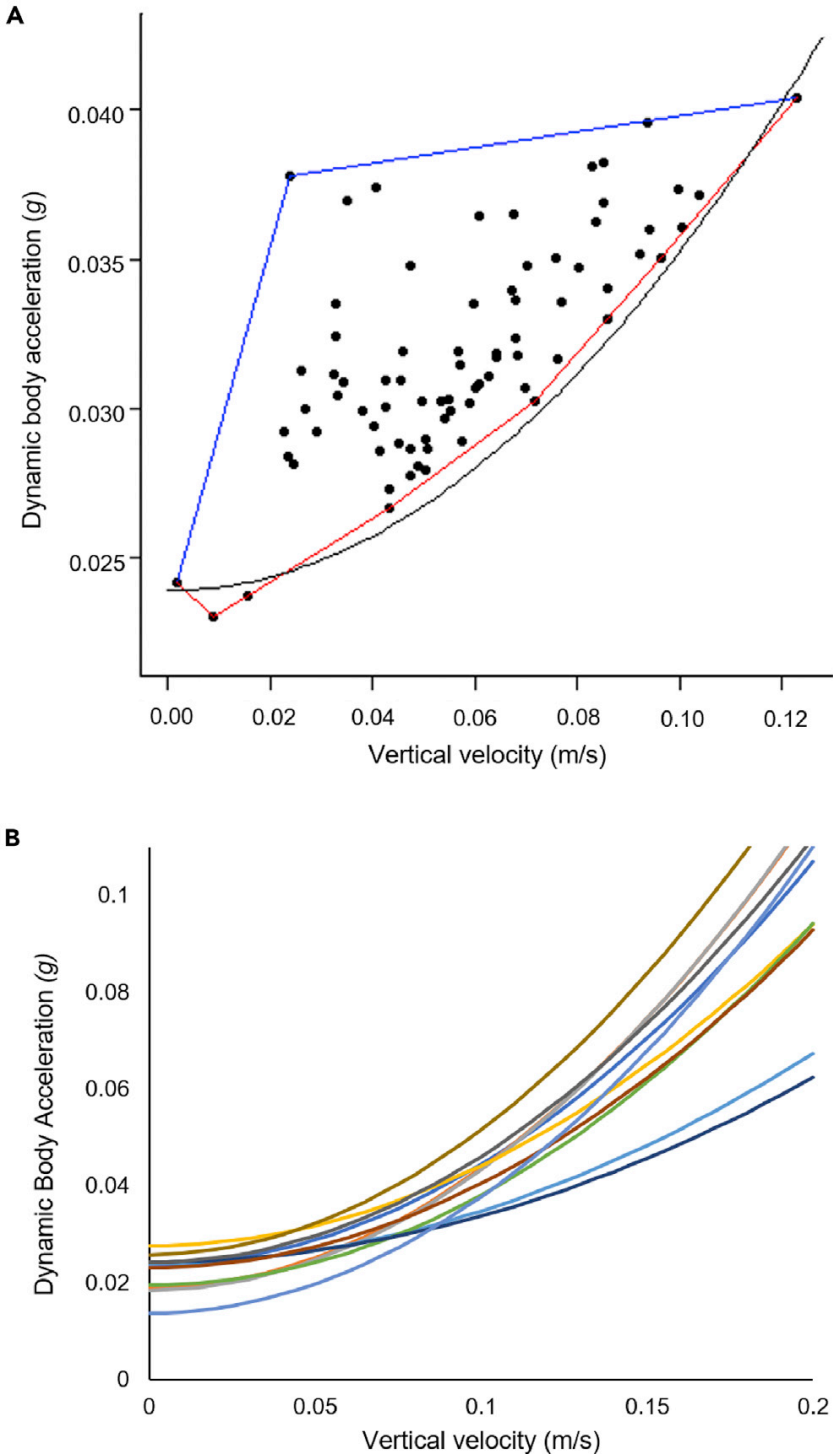
Minimum proxy for power curve (A) Calculating the minimum proxy for power curve: Dots represent measured DBA versus vertical velocity for a single whale-shark. The convex hull is shown in blue and red. The red part is the bottom half of the convex hull. The minimum power relationships are obtained by fitting a quadratic curve of the form DBA = a+b∗vertical velocity^2^ (black line) to the vertices of this red curve. (B) A series of minimum proxy for power fits from the different whale sharks (depicted by different colors) showing inter-individual variability.

These relationships were visually clearest (Figure 1) if surface swimming (depths of <3 m) were excluded. These were higher than those of sharks swimming horizontally at depth, probably because the dorsal fin-attached tag was subject to wave and chop action (see Watanabe et al., 2019), artificially elevating the DBA signal owing to environmental forces (Wilson et al., 2020a). Overall, the use of our defined convex hull method (Figure 2A) gave excellent curve fits (all r^2^–values >0.7) for the lower boundary of the DBA versus vertical velocity curve according to DBA = a+b.vertical velocity^2^ although there was an appreciable variation between individuals (Figure 2B). Other fits, such as exponential, gave lower r^2^–values, which we judged less appropriate as we wished to be maximally representative in our descriptors of how the various multiple factors that result in energy expenditure with changing ascent angles (see later in discussion) relate to vertical velocity.

Changing the confidence interval to remove outliers (by changing the relevant p-level - see Figure S1) had minimal effect.

**Figure 3:**
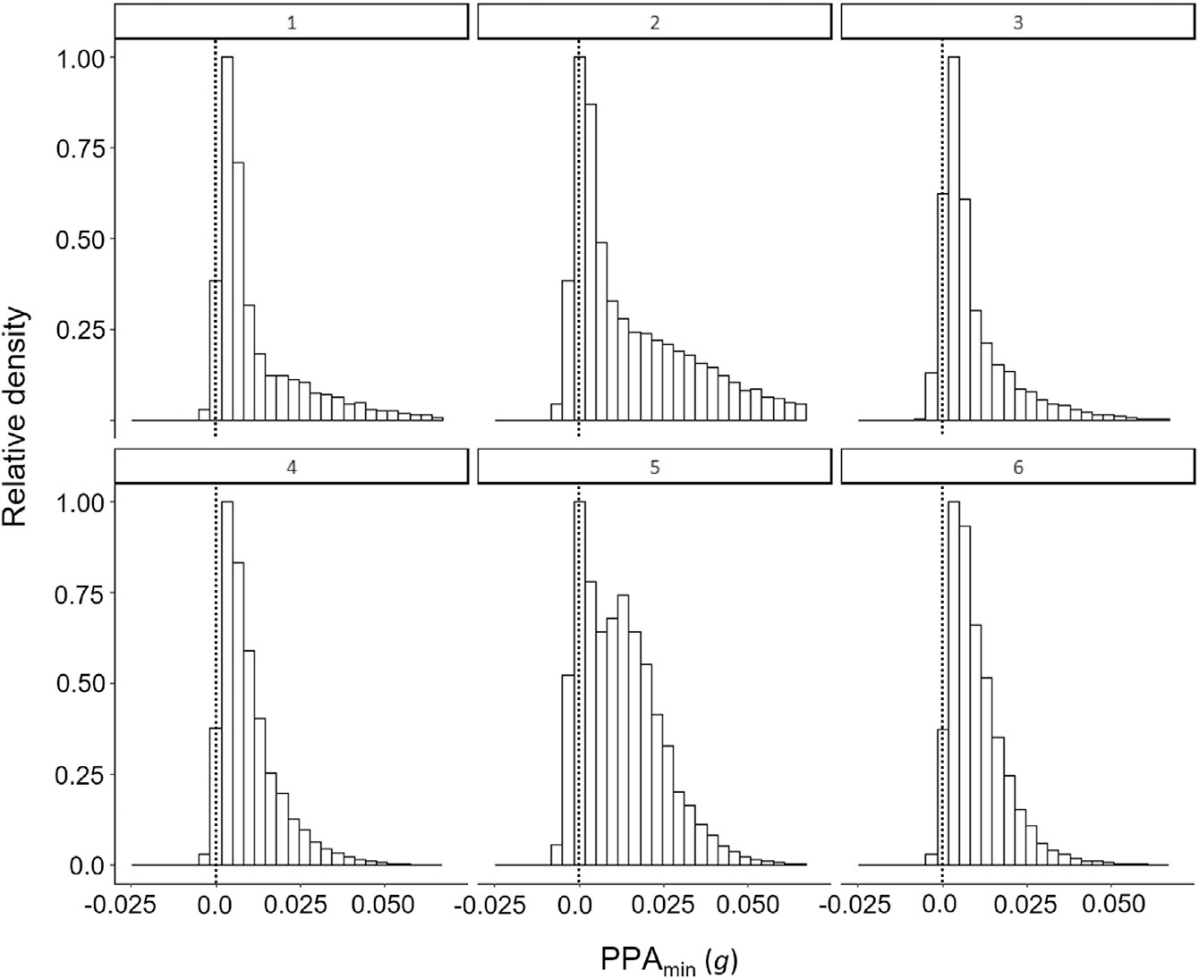
Frequency distributions Example plots of frequency distributions of the proxy for power above the minimum (PPA_min_) for six individual whale sharks (ID shown above each graph) showing inter-individual variation.

Calculation of PPA_min_ across whale sharks generally gave right-skewed frequency distributions with monomodality (Figure 3) (although bimodality also occurred (e.g., individual 5 in Figure 3). However, there was appreciable inter-individual variation (Figure 3). Further examination of PPA_min_ values substantially higher than zero within the whale shark data indicated a variety of drivers of this, including increases in traveling speed/acceleration while swimming horizontally (e.g., at the surface) (Figure 5A) as well as during the descent phases of dives (Figure 5B) and substantive changes in overall body motion owing to e.g., gill-clearing behaviors (analogous to coughing) without the sharks apparently changing speed (Figure 5C). This latter behavior was verified from video recorded by the on-animal camera included in the tag package (Video S1).

Video S1. Gill-clearing by whale shark

PPA_min_ metrics were also higher during behaviors that we assumed were related to feeding because, at this time, sharks typically swam in circles as they have been observed to do during feeding (Cade et al., 2020; Taylor, 2007) (Figure 6).

## Discussion

The concept of using PPA_min_ as a metric for highlighting power-use beyond the minimum required during movement has gained traction through this work, but its value needs careful consideration. For example, we noted appreciable inter-individual variability in the DBA_min_ versus vertical velocity curves (Figure 2B), which may be owing to DBA variation with activity as a function of shark size (cf. Gleiss et al., 2013; Martin Lopez et al., 2022), inter-individual variability anyway, and/or owing to the way the tags were attached (because differing attachment methods and sites have been shown to affect acceleration metrics, including DBA (Wilson et al., 2020a). Although all sharks were fitted with tags using the same attachment mechanism, the position of the tag within the range of the height of the dorsal fin could not be reliably standardized. This meant that the precise position may have led to variable movement in the fin itself. In addition, because the attachment mechanism was spring-based, there may have been a more or less stable attachment depending on how the spring reacted to the variable fin thickness at the site of attachment. Ultimately though, variation between individuals in the DBA_min_ versus vertical velocity curves should be unproblematic provided that there is enough information on DBA at a variety of vertical velocities recorded from the study animals to construct the curves.

Choice of the curve describing the proxy for minimum power is critical in the determination of PPA_min_. We used 95% lower limits in our plots of DBA versus vertical velocity to remove outliers for whale sharks owing to, for example, noise in the data-gathering technology, noting that other limits made little difference in the quadratic term used in the line equation (Figure S1). This, however, raises the question as to why animals should be displaying power proxies below the minimum at all (Figure 3). Examination of data shows that this was always owing to periods where the sharks momentarily glided up using less power than predicted for a given vertical velocity owing to them momentarily benefitting from their momentum (which is considerable in whale sharks as momentum is the mass multiplied by the velocity). The likelihood that this occurs can be reduced by selecting longer periods over which to extract DBA against vertical velocity data (see STAR Methods) although these should not be so long as to incur great variation in either. Otherwise, some shark species sometimes use upwelling areas to minimize power to swim (Papastamatiou et al., 2021), which would allow a reduction in DBA. This seems to be a highly localized phenomenon for marine animals although it may be considered in some birds (see later in Discussion) (Williams et al., 2020). Certainly, consideration of the energy landscape (Shepard et al., 2013) is needed to account for particularly low DBA values during traveling.

The utility of PPA_min_ lies in our selection of the main power-modulating factor in the study of animal travel. Sharks, like birds, have a higher density than the medium in which they move so the rate of change in depth, or height, involves a corresponding change in potential energy which is why animal power is involved (Pennycuick, 2008). This explains why the rate of change in depth is also viable as a parameter on which to base PPA_min_. Importantly, sharks can choose to vary their body angle and vertical velocity across a wide range (e.g., Figure 1) and for various reasons, but they are physically constrained to provide minimum energy for locomotion accordingly. Thus, PPA_min_ does not highlight either body angle or vertical velocity as parameters of interest *per se*. Instead, it simply highlights when the power used for travel, including at various vertical velocities, is greater than the minimum.

**Figure 4:**
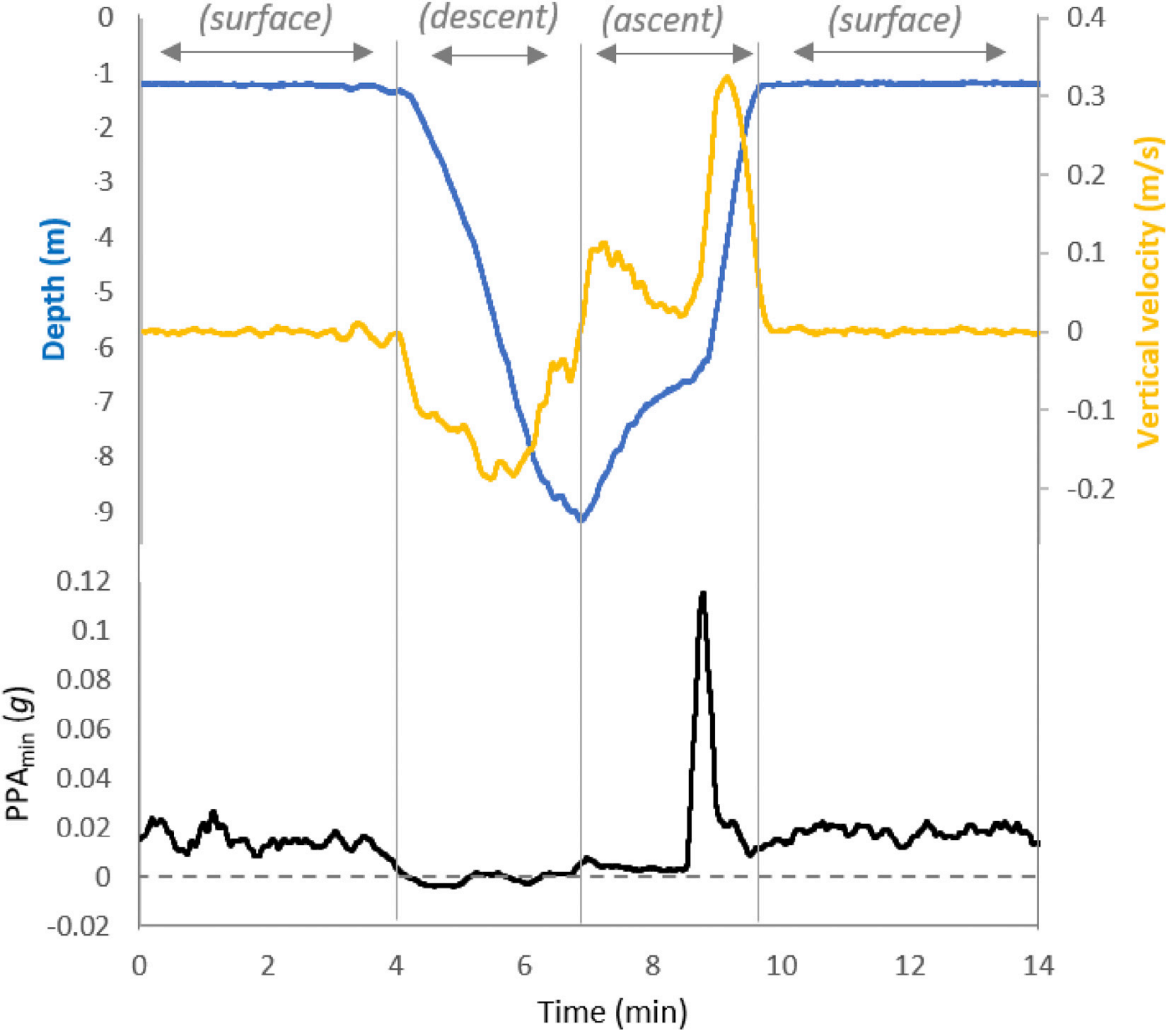
PPA_min_ at different depths A dive by a whale shark between periods swimming at the surface, showing depth (blue line) and vertical velocity (yellow line) and the proxy for power above the minimum (PPA_min_) (black line). Note how the periods at the surface have high PPA_min_ values, that the descent has a PPA_min_ that is close to 0 g, and that the ascent has a PPA_min_ that is predominantly close to 0 g except for a notable peak at around 6 m depth when the shark suddenly increased its vertical velocity. This peak was presumably owing to the investment of power required to accelerate the shark quickly from the lower to the higher vertical velocity.

**Figure 5:**
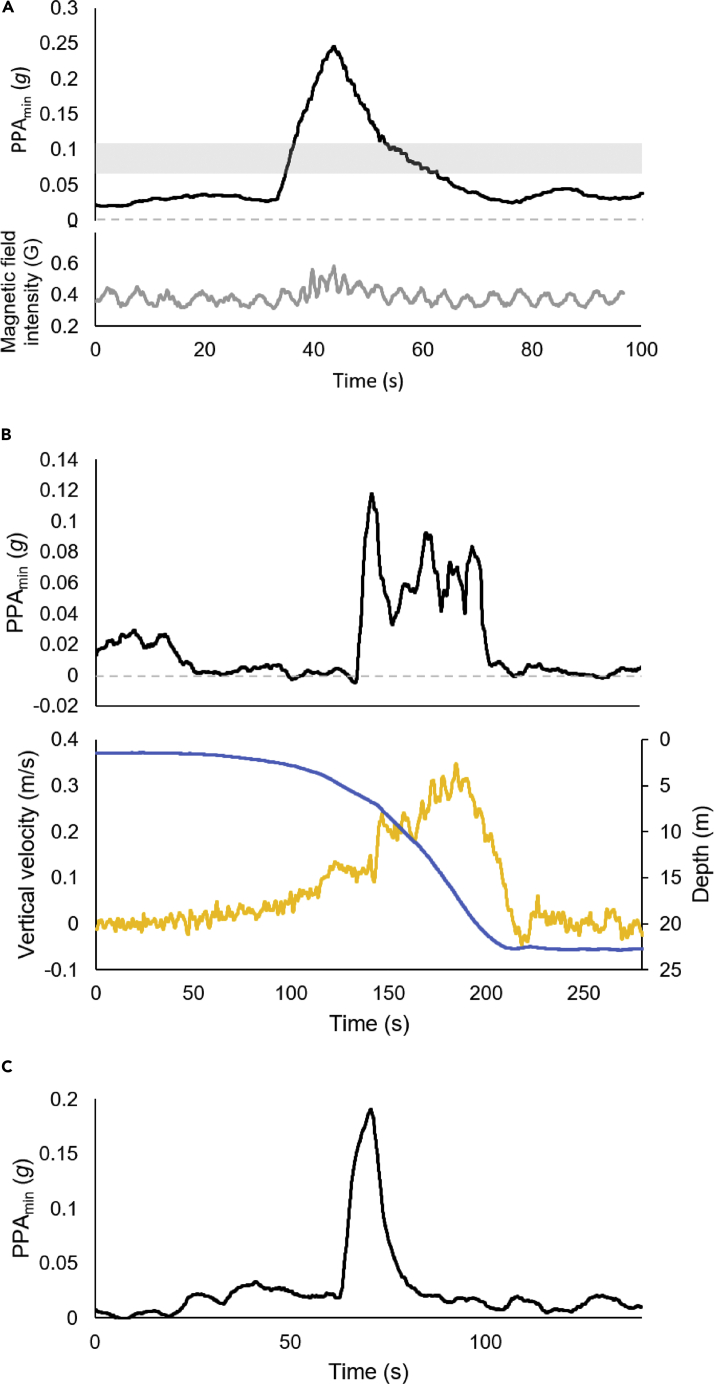
PPA_min_ during different swimming behaviors Substantial increases in the proxy for power above the minimum (PPA_min_) (black lines) in; (A) a whale shark swimming horizontally at the surface at a constant depth (ca. 1.1 m) and suddenly increasing tail-beat frequency (and presumably speed) - illustrated by the magnetic field intensity in the z axis of the magnetometer (gray line - the gray bar shows the normal range of PPA_min_ values for surface-swimming whale sharks), (B) a whale shark diving (depth shown by the blue line) with increasing vertical velocities (yellow line) but showing an abrupt transition between PPA_min_ values around zero to a maximum of >0.12 g and a plateau around 0.06 g lasting around 70 s, and (C) elevated but transient increase in PPA_min_ in a whale shark swimming horizontally near the surface (depth ca. 1.5 m) owing to the animal engaging in gill-clearing (verified via Video S1). No change in depth or tailbeat frequency was apparent during this period although there were very obvious increases in body motion (see Video S1).

As the power used to move in water increases with the cube of the velocity (Altringham and Johnston, 1990), PPA_min_ should be particularly sensitive to speed. This is what we observed during periods of horizontal swimming near the water surface (Figure 4) where speeds seem unusually high as judged by the increase in PPA_min_ over that incurred during descents of ascents (Figure 4), a phenomenon that has also been reported in other shark species (e.g., Watanabe et al., 2019). It is intriguing to speculate why. Higher speed swimming (derived by using trigonometry on body pitch and vertical velocity) was also highlighted on occasion in individual whale sharks by short-term substantial PPA_min_ values both for descents (e.g., Figure 5B) and ascents although the reasons for them doing this are unclear. We speculate that the animals were either reacting to vessels or other perceived threats and inspection of where these events occurred may prove useful for conservation reasons (Womersley et al., 2022).

Recent work on oceanic whitetip sharks *Carcharhinus longimanus* has also examined the value of animals traveling at various speeds, and most notably COT_min_, which normally occurs at speeds higher than minimum power, and is particularly relevant for optimizing the net rate of energy gain during foraging (Papastamatiou et al., 2018). Against this, other work has noted that white sharks *Carcharodon carcharias*, for instance, travel at lower speeds than their projected COT_min_ (Watanabe et al., 2019). Given that most high PPA_min_ values are likely to be related to speed increases (but see later in discussion), consideration of these values may provide insight into optimal speeds for different species or within species exhibiting different foraging behaviors, even if speed is not measured directly (cf. Papastamatiou et al., 2018).

**Figure 6:**
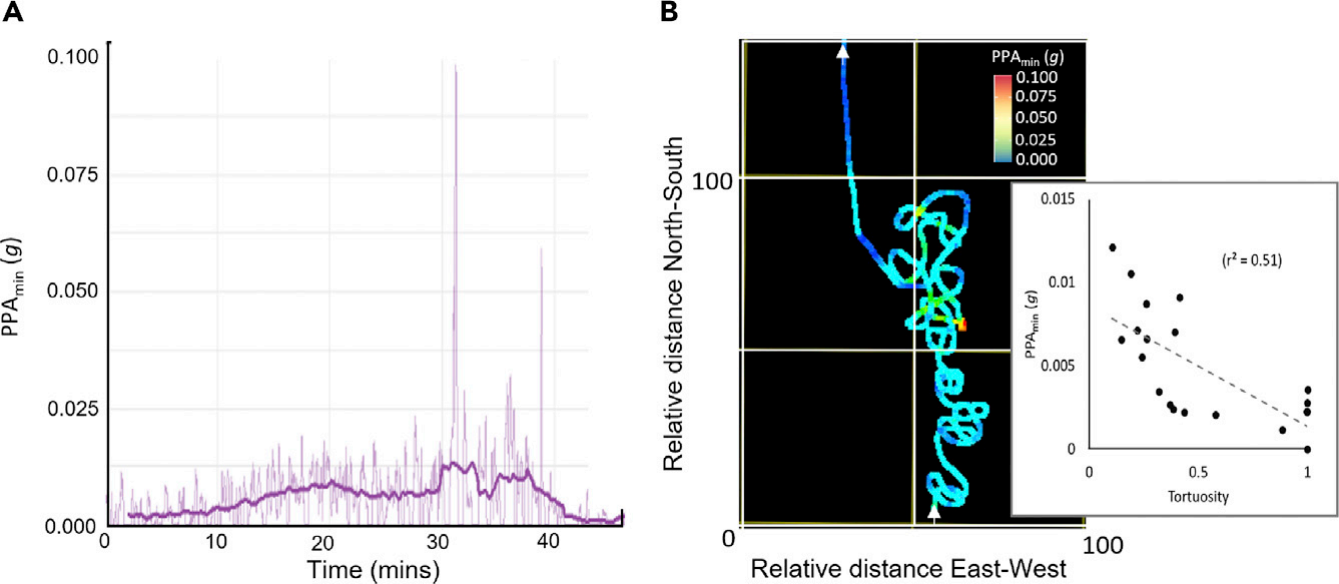
Changes in PPA_min_ related to track tortuosity (A) Changes in PPA_min_ over time in a whale shark (the thick line shows smoothed data) and (B) the 2-dimensional pseudo-trajectory (dead-reckoned using a constant speed (Gunner et al., 2021) – at a swim speed of 1 m/s, the complete track would translate to a distance of *ca*. 2.7 km) for the same period highlighting areas with high PPA_min_ values in warmer colors (the white arrows show the direction of travel from bottom to top). Note how the PPA_min_ tends to increase with track tortuosity (see inset which plots tortuosity [straight line distance over 3 min/total track length over 3 min] against PPA_min_ for the section of track shown - a value of 1 is straight line travel with increasing tortuosity represented by smaller numbers). During much of this time, the shark was assumed to be feeding and the increase in PPA_min_ presumably results partly from swimming with the increased drag of an open mouth.

Other than that, we noted that PPA_min_ highlighted transient behaviors such as gill-clearing and that increases in PPA_min_ were also associated with behavior believed to be feeding. This was evidenced by swimming in circles (Figure 6), which can occur when whale sharks are repeatedly passing through prey patches (Cade et al., 2020). Here, the increases in PPA_min_ are presumably owing to increases in power associated with swimming with the mouth open, which will increase the drag (Cade et al., 2020). This suggests that increases in PPA_min_ may highlight feeding at times other than circling if marked increases in swim speed can be precluded.

Aside from short-term instances of raised PPA_min_, we note the great inter-individual variability in PPA_min_ (Figure 3) which may be longer term adherence to different power regimes or may be owing to different signal strengths relating to animal size or differences in the attachment site and stability. Within any one animal’s data though, generally raised PPA_min_ over longer periods may highlight periods of concern for conservationists, particularly for species that operate on an energetic knife edge (Sims, 1999) because small changes in energy outgoings can have disproportionate consequences in these animals (Gorman et al., 1998). Otherwise, regressions of generally wider DBA versus other power-dependent parameters of interest could be a useful indicator of species lifestyles. For example, we would expect more active species (such as pursuit predators) to exhibit generally higher PPA_min_ than less active species such as whale sharks, because they have a more dynamic lifestyle and operate over wider speed ranges (cf. Meese and Lowe, 2020).

In this work, we have only highlighted some of the potential of the PPA_min_ approach for helping determine how whale sharks allocate their energy to time during traveling. There are obvious conservation ramifications for this, such as how whale sharks may react to ecotourism. Whether DBA can be used to determine the minimum costs of travel remains to be seen but, in the meantime, we suggest that considering PPA_min_ across a broader range of species may prove fruitful for helping to understand when, how, and why animals move using more power than they apparently need. We expect this to change with species lifestyle but also according to circumstances that vary over time. This may be relevant for issues that range from cases where increased haste is beneficial (predator avoidance, prey acquisition, movement through threatening environments) to changes in the frequency or intensity of transient behaviors that occur during movement and ultimately help us assess drivers of energy use at a time when environments are changing so rapidly.

### Limitations of study

The ability to determine the minimum power line depends critically on having enough data to construct it. This is primarily dependent on two things; simply having enough data (so animals need to be tracked for an appropriate period), and the extent to which the animal in question tends to move using minimum power. Inspection of the whale shark data indicates that they do seem to move at minimum power for much of their time, as evidenced by the accumulation of points along the bottom line (this is also expected given their low-energy lifestyle), but other animals may have a distribution that sits away from the line so that much longer periods are needed to construct the minimum power line with any confidence.

Many factors change the use of power in moving animals, including speed and change in potential energy in animals traveling in fluid media (water or air), as discussed here. However, these would expand out to what has been termed the “energy landscape” (Shepard et al., 2013) if this approach were to be tried in terrestrial animals. To attempt to determine PPA_min_ in terrestrial animals would be particularly challenging, and may be impossible, because many features of the environment such as slope, substrate penetrability and superstrate characteristics (e.g., vegetation on the substrate) cannot easily be accommodated by DBA metrics (e.g., Bidder et al., 2012) and yet are known to play a large role in modulating power to move.

## STAR★Methods

### Key resources table

**Table:** 

REAGENT or RESOURCE	SOURCE	IDENTIFIER
**Deposited data**		

pitch vs DBA data	This paper	Figshare (https://figshare.com/s/34fb3b0d770b87d74b2a)

**Software and algorithms**		

RStudio	This paper	RStudio (https://www.rstudio.com/
DDMT	This paper	Wildbyte Technology (http://wildbytetechnologies.com)

**Other**		

Daily Diary tags	This paper	Wildbyte Technology (http://wildbytetechnologies.com)
VEMCO receiver and directional hydrophone	This paper	Innovasea (https://www.innovasea.com)
VEMCO V16 continuous acoustic transmitter (https://www.innovasea.com)	This paper	Innovasea (https://www.innovasea.com)
Paralenz on-animal video camera (https://www.paralenz.com/)	This paper	Paralenz (https://www.paralenz.com/)

### Resource availability

#### Lead contact

Further information and requests for resources and reagents should be directed to and will be fulfilled by the lead contact, Rory P. Wilson (r.p.wilson@swansea.ac.uk).

#### Materials availability

This study did not generate new unique reagents.

### Experimental model and subject details

This research was conducted under Western Australian Department of Biodiversity Conservation and Attractions Licence No. FO25000033-9, FO25000033-16, CE006122 and CE006335, and Animal Ethics Approvals from The University of Queensland (Permit No. SBS/085/18/WA/INTERNATIONAL) and Murdoch University (Permit No. RW3327/21).

Data used in this study are from twelve Daily Diary (DD) tags (Wildbyte Technologies http://wildbytetechnologies.com/tags.html) (Wilson et al., 2008) that were deployed on whale sharks (*R. typus*) at Ningaloo Reef, Western Australia. The DDs were powered by a Saft 3.6 V Lithium battery and measured tri-axial acceleration (20 Hz), tri-axial magnetometry (13 Hz), pressure and temperature (4 Hz) with 16 bit resolution, recording data onto a 2 Gb memory card. The electronics were sealed inside a water-tight turned housing with O-ring seal. The unit measured 20 cm in length. Tags were attached to a custom-made metal spring clamp (similar to that described in Gleiss et al. (2009)). Ten deployments also included a VEMCO V16 continuous acoustic transmitter (https://www.innovasea.com) and a Paralenz on-animal video camera (https://www.paralenz.com/) on the clamp.

### Method details

At Ningaloo Reef, Western Australia, a spotter plane was used to locate whale sharks swimming at the surface. The pilot then directed the research vessel to a position approximately 50 m ahead of the shark and a researcher entered the water and, while swimming alongside, deployed the tag package on the shark’s first dorsal fin. A VEMCO VR100 acoustic receiver and directional hydrophone (https://www.innovasea.com) onboard the research vessel were used to track 10 of the tagged sharks and allow retrieval of the DD after ∼ 2–5 h, while the other two tags were left in place for a period of days before being recovered when the sharks were resighted.

### Quantification and statistical analysis

Tag data were assessed with DDMT (Wildbyte Technologies http://wildbytetechnologies.com), a program that *inter alia* displays all data channels in the same time-line and allows workers to use an automated Boolean-based approach for identifying behaviors (Wilson et al., 2018). Using this, periods of swimming were identified during which swim angle did not vary by more than 2° over at least 10 s. For these periods, data on start and end depth and the duration of the event were extracted to derive vertical velocity (rate of change of depth) as well as the mean smoothed (over 5 s) vectorial dynamic body acceleration (the DBA metric) (Wilson et al., 2020a). Vectorial dynamic body acceleration (VeDBA) is the dynamic component of the tri-axial acceleration signal provided by orthogonal sensors recording at high frequencies (>10 Hz) (Wilson et al., 2020a). It is calculated by subtracting the smoothed acceleration (over 2 s (Shepard et al., 2008)) of each acceleration axis from its raw acceleration before adding the components of the vector (Qasem et al., 2012).

The power for a shark to move up the water column should ostensibly predominantly relate directly to the rate of change of gravitational potential energy, which is given by *m.g.∂d/dt*, where *m* is the mass, *g* is the gravitational constant and *∂d/dt* is the rate of change of depth. The expectation from this is that minimum (power) DBA values for sharks ascending the water column should scale linearly with vertical velocity. However, fish are assumed to incur an energetic cost due to the energy required to perform their lateral body undulations (Bale et al., 2014) in addition to incurring different amounts of drag according to the angle of attack (including the angle of attack of their bodies), which would lead to correspondingly varying thrust (and therefore propulsive power). This explains why our data, and data presented in Gleiss et al. (2011), show a clear non-linear relationship between both tri-axial and biaxial dynamic body acceleration and body pitch. Further, the complexity of the number of factors affecting the relationship between DBA and vertical velocity would make descriptors that would perform well with simple mechanistic explanations, such as exponential relationships, less likely to be as good as quadratic fits. As a result, we examined descriptors for DBA versus vertical velocity with quadratic fits, which were, as predicted, markedly better than exponential fits. For this, we used data for each individual shark to examine how a proxy for power related to vertical velocity with a view to defining minimum (proxy for) power as a function of vertical velocity. We began by removing all the datapoints where the vertical velocity was less than zero, as the minimum power required for sharks to move downwards is zero (because they are denser than water). Our aim was to find a relationship providing a mathematical descriptor that more-or-less “sits under” the remaining points, but excluding any outliers that are likely to be due to noise in the data. This relationship would then define the estimated minimum proxy for power for each animal in relation to vertical velocity. Any periods where the vertical velocity was less than zero were allocated the mathematical value for the VeDBA at a vertical velocity of zero (for reasons stated above) from this relationship. To construct this relationship, we started by finding, for each individual, the line of best fit to all the data where the vertical velocity was >0 m/s. We then removed all points that were outside the 95% confidence intervals of this fit (i.e., removing outliers) and found the convex hull of the remaining points. The convex hull consists of two piecewise-linear curves joining the leftmost point to the rightmost point, one of which lies above the other. We discarded the top curve and fitted a quadratic function of the form DBA = a+b∗vertical velocity^2^ to the vertices of the bottom curve. This model describes the minimum proxy for power increment with vertical velocity (Figure 2A). To calculate the proxy for power above the minimum (PPA_min_) used by sharks across their tagged periods, we extracted their vertical velocities over time and used our proxy for minimum power curves to calculate the minimum power across time. These values were then subtracted from the corresponding smoothed DBA values over time to give PPA_min_.

## Data Availability

• All data reported in this paper will be shared by the lead contact upon request. • This paper does not report original code. • The pitch vs DBA data are archived in Figshare (https://figshare.com/s/34fb3b0d770b87d74b2a). Any additional information required to reanalyze the data reported in this paper is available from the lead contact upon request.
