# Supplementary material for: Highlighting when animals expend excessive energy for travel using dynamic body acceleration
Source: iScience. 2022 Aug 24;25(9):105008. doi: 10.1016/j.isci.2022.105008 (PMC9464956; doi:10.1016/j.isci.2022.105008)
Supplement: Document S1. Figure S1 [file mmc1.pdf]

iScience, Volume *25*

Supplemental information

Highlighting when animals expend excessive energy for travel using dynamic body acceleration

Rory P. Wilson, Samantha D. Reynolds, Jonathan R. Potts, James Redcliffe, Mark Holton, Abi Buxton, Kayleigh Rose, and Bradley M. Norman

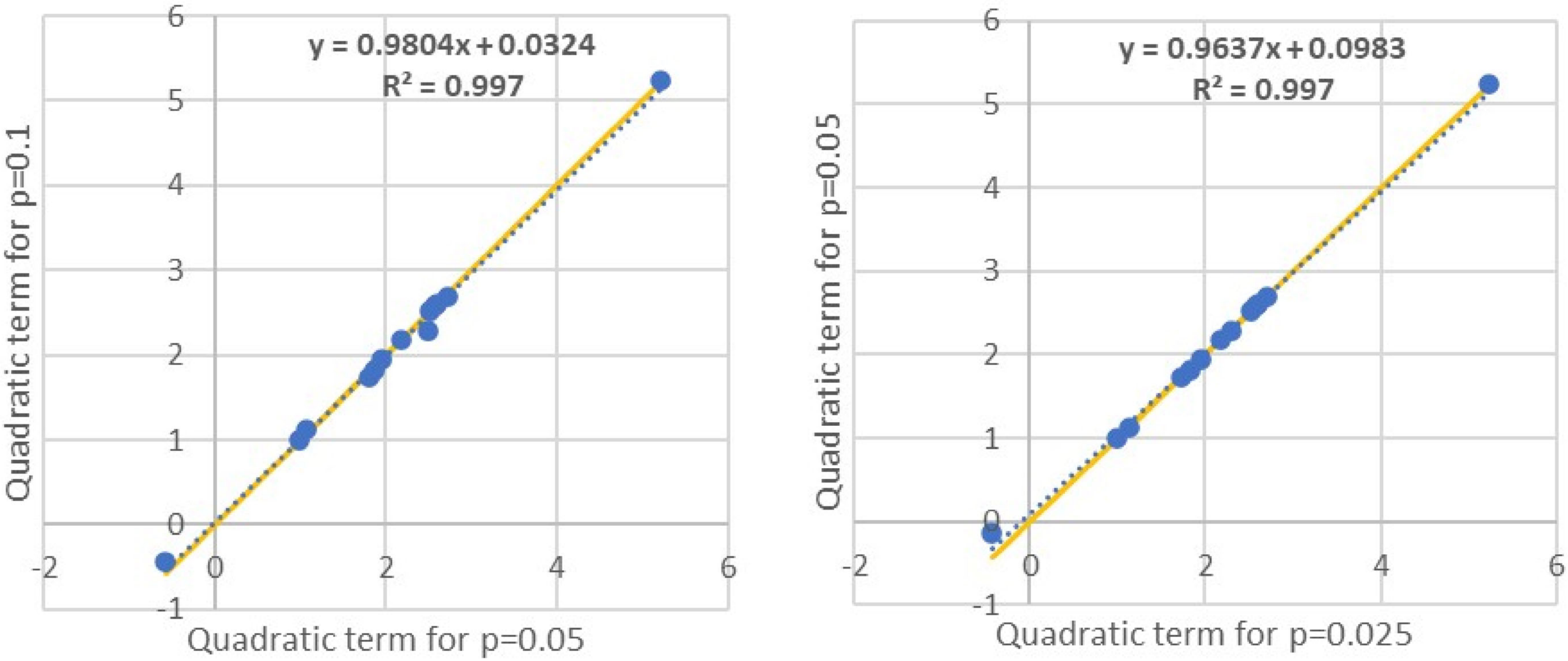

Figure S1. Confidence interval adjustment (related to Figure 2)
